# Effect of Surgical Margins on Local Recurrence Patterns of Myxofibrosarcomas: A Retrospective Cohort Study From the Stockholm Sarcoma Centre

**DOI:** 10.1002/jso.70076

**Published:** 2025-09-03

**Authors:** Madeleine N. Hoang, Pavlos Doumanidis, Emmy Nyqvist, Jenny Löfgren, Asle C. Hesla, Panagiotis Tsagkozis

**Affiliations:** ^1^ Department of Molecular Medicine and Surgery Karolinska Institute Stockholm Sweden; ^2^ Department of Orthopaedic Surgery Karolinska University Hospital Stockholm Sweden; ^3^ Department of Reconstructive Plastic Surgery Karolinska University Hospital Stockholm Sweden

**Keywords:** margins of excision, neoplasm recurrence local, radiotherapy, sarcoma

## Abstract

**Introduction:**

One of the most common subgroups of soft tissue sarcomas are myxofibrosarcomas (MFS), which possess an infiltrative and aggressive growth pattern with a tendency for local recurrence (LR). MFS presents significant management challenges due to difficulties in achieving satisfactory surgical margins. The present study describes the LR pattern of MFS and its association with resection margin, re‐excision surgery and adjuvant radiotherapy.

**Method:**

This is a registry study conducted at the Sarcoma Centre at Karolinska University Hospital, Stockholm. All patients who underwent surgery with curative aim for MFS in this centre between 2010 and 2023 were included.

**Results:**

LR was documented in 17/87 patients. Surgical margin was predictive of LR but not overall patient survival. The cumulative LR incidence at 3.5 years for intralesional, marginal, and wide margins were 80%, 40% and 10% respectively. RT was not shown to confer any suppressive effect on LR. Amputation was needed in five patients for local disease control.

**Conclusion:**

Margins are the foremost determinants of LR for MFS. Proactive surgical management should be considered when margins are marginal.

## 1 Introduction

Myxofibrosarcoma (MFS) is an entity of soft tissue sarcomas (STS) which commonly arises in the limbs of elderly patients and is described as a painless, indolent, largely nodular tumour [1]. Possessing an invasive growth pattern, MFS will typically extend along the fascial planes and into surrounding tissues. Boundaries are often difficult to assess intraoperatively due to microscopic infiltration, resulting in a high propensity for local recurrence (LR) ranging from 14% to 60% [2, 3, 4, 5, 6, 7, 8, 9]. Surgical margin has been described as an independent predictor of LR while tumour characteristics are reported as determinants of overall survival [10]. Treatment modalities available for sarcoma consist of surgery, radio‐ and chemotherapy. In the setting of MFS, the optimal resection margin has yet to be defined, and the role of radiotherapy (RT) is uncertain.

Historically, various classification systems have been employed to describe surgical radicality. The Enneking staging system has grades ranging from intralesional, marginal, wide, to radical. According to Enneking, an intralesional margin is defined as a resection plane engaging the tumour. Marginal resections are clear of neoplastic tissue but have entered the reactive zone, while a wide resection demands a cuff of normal tissue [11]. Margin quality is evaluated based on tissue composition, which is believed to influence its effectiveness as a boundary [8]. A study focusing on high‐grade, infiltrative tumours by Fujiwara et al. highlighted that good quality of surrounding tissues acting as barriers may be critical in local control [12]. In their study, the sturdy properties of fascial structures and periosteum served adequately as confinement, as patients did not present with a higher rate of LR despite narrower resection margins. A large tumour may be difficult to resect causing a substantial risk of LR along with the high likelihood of metastasis associated with sizeable tumours. The overall amputation rate for sarcoma is around 5% thanks to advances in oncosurgical techniques. For high‐grade MFS, rates up to 17% have been reported [13].

The role of adjuvant RT in case of positive margins is unclear; while some argue that RT promotes tumour control, others assert that no additional benefit is provided [13, 14]. Current recommendations from the European Society for Medical Oncology (ESMO) state that patients with MFS derive the greatest therapeutic effect from RT compared to other subtypes [15, 16]. The present study sets out to primarily examine the relationship between margin, local disease recurrence, and overall survival. The secondary aim is to describe any prognostic factors that may influence the outcome for patients with MFS.

## 2 Materials and Methods

This is a registry‐based study combined with a patient record review of patients who received surgery for histopathologically verified MFS between November 2010 and September 2023 at the Stockholm Sarcoma Centre. Eligibility criteria included curative intent and an age of 18 years or older. Variables detailing demographic information and tumour characteristics were extracted from the national sarcoma registry with any missing data complemented through review of patient records. Data were extracted from the Swedish national sarcoma registry in September 2023.

### 2.1 Patient Data

The electronic medical records of 91 patients with MFS were examined. Of them, 87 received surgical treatment, with 12 (14%) having previous surgery as an unplanned procedure. Age, sex, tumour grade and size, anatomical location, and surgical margins were documented (Table 1).

**Table 1:** Baseline characteristics.

Demographics and tumour characteristics	*n* (%)	*p*‐value
Patients	87	
Sex		
Male	55 (63)	
Female	32 (37)	
Age at diagnosis, mean ± SD	69.5 ± 12.29	
Tumour size ± SD	6.8 ± 4.9	
Tumour depth		
Superficial^a^	61 (70)	
Deep	26 (30)	
Grade^b^, FNCLCC		
Low grade	19 (21)	
High grade	67 (77)	
Location		
Lower leg	28 (32)	
Thigh	22 (25)	
Lower arm	9 (10)	
Other	28 (32)	
Surgical margin		
R0	60 (69)	
Wide	30 (34.5)	
Marginal	30 (34.5)	
R1, Intralesional	27 (31)	
Oncological therapy		
Radiotherapy (RT)	34 (39)	
RT + chemotherapy	6 (7)	
Dosage		
Gy < 50	4 (12)	
Gy ≥ 50	30 (88)	
Site of primary surgery		
Sarcoma centre	75 (86)	
Non‐sarcoma centre	12 (14)	
Local recurrence	17 (19.5)	
Number of surgeries, mean (range)		
Cumulative sarcoma surgeries^c^		
Unplanned excisions	1.67 (1–4)	< 0.01
Planned excisions	0.55 (0–4)	
Irrespective of cause^d^		
Unplanned excisions	2.67 (2–5)	
Planned excisions	1.58 (1–5)	
Follow‐up (months)	65 ± 42	

*Note:* Percentages are presented in brackets.

^a^ Subfascial denotes a location above the fascial plane, i.e., subcutaneous lesions

^b^ High grade equal to grades 2 and above.

^c^ Including index surgery, recurrence surgery and expanded resections.

^d^ Including index surgery, recurrence operations, expanded resections, surgery for complications, and reconstructive surgery.

The mean follow‐up period was 42 months (ranging from 1 to 103 months). For high‐grade tumours, postoperative follow‐up at the outpatient department was conducted every 3 months for the first 2 years, every 6 months for the following 3 years and annually for another 5 years. For low‐grade tumours, postoperative routine follow‐up evaluation was performed every 6 months for the first 5 years, and then annually for another 5 years. Chest imaging and a physical examination of the operated area were part of the follow‐up assessment. Postoperative oncological adjuvants and follow‐up regime was decided at a multidisciplinary musculoskeletal tumour meeting. Adjuvant radiotherapy was generally offered to high‐grade tumours, for all deep‐seated as well as for superficially excised lesions with a marginal or intralesional margin. Standard postoperative radiation dosage was 50–60 Gy. The day of the operation served as the start of the follow‐up, and the date of the patient's death or the last follow‐up was the end of the follow‐up. Margin definitions were extracted from histopathological reports performed by experienced sarcoma pathologists at our centre. For cases of previous unplanned surgery, the margins acquired at re‐excision became the effective verdict.

### 2.2 Statistical Analysis

All statistical analyses were performed using R (version 4.3.1). Pearson's chi‐square (*χ* ^2^) test was used for comparisons of categorical variables between groups while continuous variables were analysed with Students *t*‐test. Survival analysis was done using the Kaplan–Meier method and log‐rank test was used for comparisons between groups. A swimmer plot was generated with the ggplot2 package to visually present recurrence events. All tests were double‐sided, and a *p*‐value of < 0.05 was considered statistically significant.

### 2.3 Ethical Considerations

An ethical permit was granted from the Swedish Ethical Review Authority with permit number 2022‐03044‐01. This study was carried out in accordance with the Declaration of Helsinki's criteria.

## 3 Results

### 3.1 Descriptive Characteristics

Among 87 patients, 55 (63%) were of male sex with a median age at diagnosis of 70 years (Table 1). Frequently affected locations included the lower limb, with the lower leg being the most common site (32%), followed by the thigh (25%) and lower arm (10%). Sixty‐one lesions (70%) were superficial and 67 (77%) were high‐grade, predominantly grade 2 (Table 1).

### 3.2 Surgical Treatment and Postoperative Radiotherapy

R0 resection was achieved in 60 patients (69%), with half reaching wide margins and the other being marginal. Intralesional excisions were documented in 27 (31%) cases in the histopathology report. Of the 27 cases of intralesional margins, 20 were planned and 7 were unplanned excisions. Intraoperatively, surgeons classified one case as intralesional, 14 as marginal, and 70 as wide.

One‐third (*n* = 34) received radiotherapy (RT) administered to 10/27 intralesional, 18/30 marginal, and 6/30 wide margins. Six patients were subjects of combined RT and chemotherapy (7%). Three (3%) were amputated at primary surgery due to a tumour location proximal to vital structures.

### 3.3 Re‐Excisions and Adjuvant Radiotherapy

Re‐excisions were offered to 10 patients with intralesional margins. Among the remaining 17 patients who did not undergo re‐excision, three received RT in favour of surgery; seven of them would go on to develop LR, including all three subjects who had RT. Three re‐excision surgery cases were after planned excisions, and all remained free of LR at follow‐up.

### 3.4 Local Disease Control and Overall Patient Survival

12 subjects (14%) underwent their index surgery outside of a sarcoma centre, all of which were < 5 cm, and all but one subcutaneous in depth. After referral, all received re‐excisions at our facility, with three concurrently undergoing plastic reconstruction to cover the resulting large tissue defect. None developed LRs. Following re‐excision for secondary LRs, surgical site infections necessitating antibiotics occurred in seven patients (41%). The unplanned excision group had a mean of 2.7 surgeries compared to the planned surgery cohort with 1.6 operations, with inadvertent excisions bearing a significant association to number of surgeries (*p* < 0.01).

**Figure 1:**
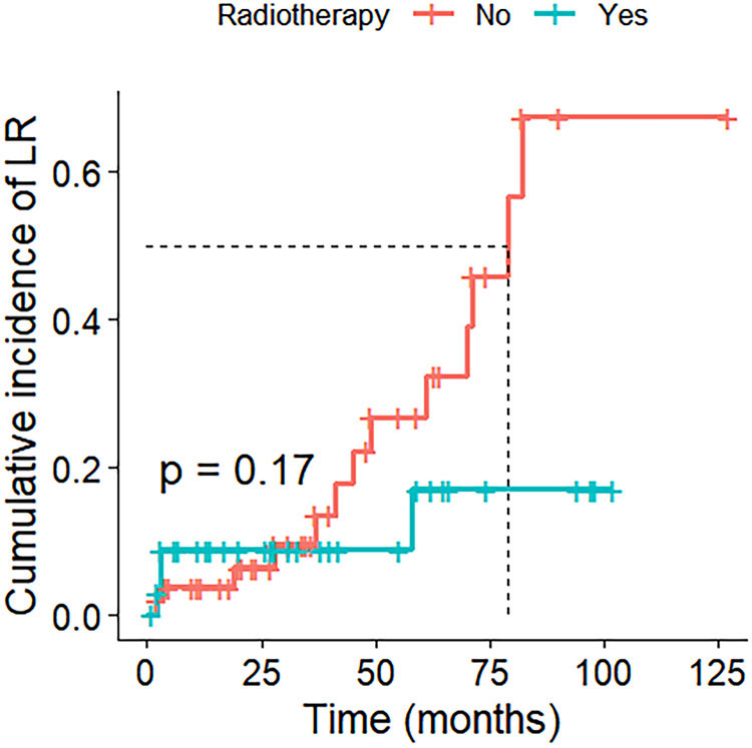
Radiotherapeutic effect on cumulative incidence of local recurrence (LR).

**Figure 2:**
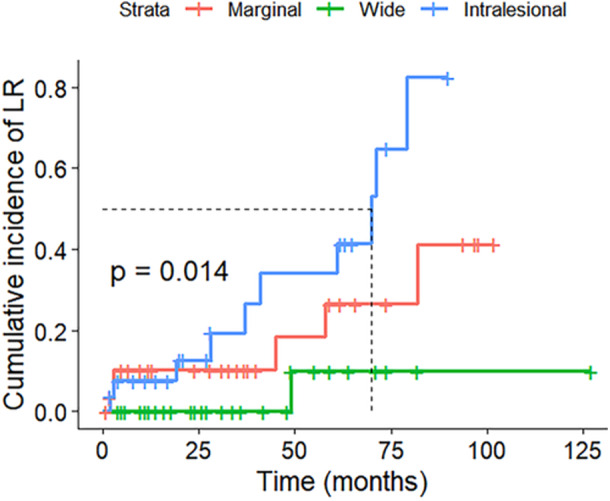
Cumulative incidence of local recurrence (LR) as a result of surgical margin.

**Figure 3:**
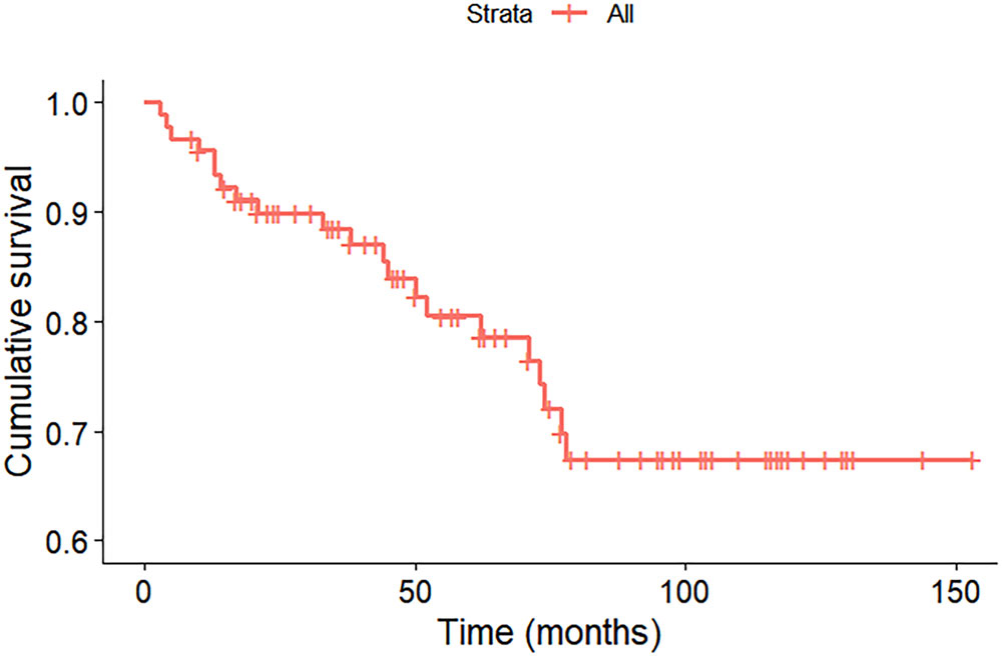
Overall survival rates.

Radiotherapy was not significantly associated with improved local control (*p* = 0.17) (Figure 1). LR was documented in 17 patients (20%) (Table 1). Surgical margin was an important factor in achieving local disease control with analysis revealing significant differences in cumulative incidence of LR between groups (*p* = 0.014) (Figure 2). Among 27 patients with acquired intralesional margins from primary surgery, 10 (37%) would go on to experience LR at some point in time. The corresponding rates for the marginal and wide margins respectively were 6 out of 30 (20%), and 1 of 30 (3%) (*p* < 0.01). Intralesional margins had an accumulative LR incidence after 3.5 years of 80%, with marginal margins reaching 40% and wide margins having 10% (Figure 3). The grade of recurrent tumours was unchanged in 12 cases. At the point of data acquisition, 21 patients (23%) had died. The 1‐, 5‐, and 10‐year overall survival rates for the 87 patients were 96%, 81%, and 67%, respectively (Figure 3).

### 3.5 Management of Recurrent Disease and Limb Survival Rate

**Figure 4:**
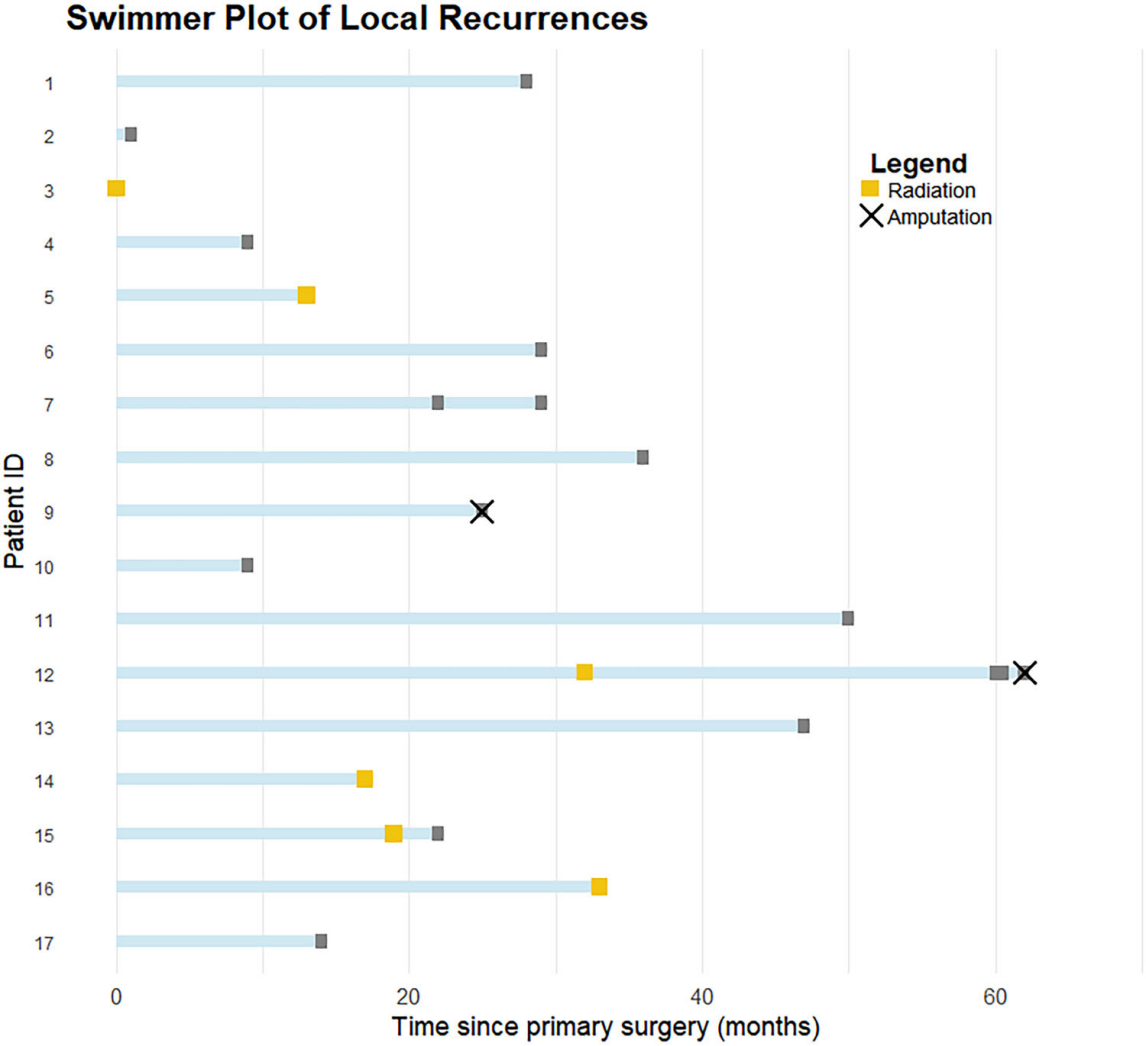
Swimmer plot of all recurrence events. Patient ID's are displayed on the *y*‐axis with time from primary surgery until recurrence on the *x*‐axis.

Of 17 LRs, 16 (18%) underwent further local excisions. These procedures resulted in five marginal margins, five wide, and six intralesional margins (Figure 4). Postoperative radiotherapy was administered to three patients Three patients had a secondary LR, one of whom developed a third LR and was subsequently limb amputated. Initially, three patients were amputated during the index operation. An additional two individuals underwent limb amputation, including the aforementioned patient, due to unsatisfactory margins and recurrences after discussion in the tumour board meeting.

## 4 Discussion

Our findings stress the importance of resection margins in acquiring local control in MFS, with RT not having demonstrated any significant role in tumour suppression.

MFS is recognised for its difficult management regarding local control of the disease [17, 18]. Although distant metastases are relatively uncommon, LR rates can reach 50%–60%, even in low‐grade tumours. With each recurrence, the grade of MFS tends to rise gradually [1, 19]. Ultimately, the tumour becomes incurable due to multiple LRs. In this study, surgical margin was found to hold an important prognostic value for LR. No major tendency for conversion to a higher grade was observed in locally recurrent disease.

Surgical excision appears to be the only effective treatment option for MFS. A significant difference in cumulative incidence of LR was shown for each margin. Marginal excisions had a high cumulative LR incidence of 40%. While marginal margins often are considered sufficient to evade LR for other STS entities, the results indicate that wide margins are required for MFS. Intraoperative macroscopic assessment of margins was often inaccurate as per the histopathological examination, further displaying the challenging nature of MFS resections. Although many of the reoperations yielded intralesional borders most were limited to one disease recurrence. This may be attributed to tumour biology rendering certain subtypes to have more favourable outcomes than others [20]. In many instances of suboptimal margins, the risk of LR was accepted to avoid further operative trauma as removal would be feasible with regular monitoring of the patient. Berner et al. reported LR rates of 14.4% by using an approach of staged excision until adequate margins were obtained, and delayed reconstruction [21]. Negative margins were obtained after a median of two surgeries. Margins have been established as important for local recurrence free survival (LRFS) but have not been proven to affect OS [6]. Similarly, overall survival was unaffected by resection margin in our study indicating that LR does not affect OS.

Opinions are divided regarding the benefit of RT. The general belief is that RT enables limb‐salvage surgery whilst providing local control through tumour cell sterilisation, especially for large and/or high‐grade MFS [14, 22, 23, 24]. In a few series, an advantage has been seen with dosages exceeding 60 Gy [25]. The Scandinavian Sarcoma Group published a study presenting 5‐year LR rates of 38% for intralesional, 19% for marginal, and 7% for wide margins in STS treated with RT and surgery [26]. In comparison, the MFS cohort of this study had cumulative LR rates of 80%, 40%, and 10% in the same order, being nearly twice as high. RT was not shown to exert any suppressive effect on tumour recurrences. Similar to this finding, a recent systematic review on MFS saw no significant associations between adjuvant RT and recurrence, metastasis, or OS [27]. On the contrary, Callegaro et al. advocate for RT in MFS management in a multicenter study showing local disease control from adjuvant therapy [17]. Some recommendations regarding neoadjuvant RT before undertaking procedures for LR have also been voiced [2].

In the present cohort, 14% had inadvertent sarcoma resections which were significantly associated with higher surgery rates, when accounting for recurrences and re‐excisions (*p* < 0.01). The same was seen when studying the total number of operations irrespective of cause, including those performed for complications or reconstructive surgery (*p* < 0.01). MFS constitute a dominant group within the unplanned excision group [28, 29]. Excisions after inadvertent surgery will usually claim larger resections [30], and in some instances limb amputation is the only viable alternative. Amputation rates due to recurrent disease lie between 3.6% and 6.5% in STS cohorts when conditions such as anticipated poor functionality after primary resection and involvement of critical structures are present [30, 31, 32]. Five amputations (6%) were conducted in total in our study group. Three were amputated during primary surgery while two were amputated on account of highly recurrent disease after repeated resections. According to our national recommendations, superficial lesions, < 5 cm may be excised without prior biopsy in non‐sarcoma centres as they are unlikely to be malignant. Due to the limited size of the study cohort, it was not possible to discern whether the status of unplanned excision was significantly associated with amputations. As commented by Smith et al. and analogous with Harati et al., there may be an inherent selection bias as cases selected for amputation oftentimes are especially intrusive [32, 33]. This does not detract from the persisting issue of unplanned excisions which contribute to a high surgical burden among sarcoma patients. Other modes of surgery need to be evaluated in future studies, such as a proactive approach when margins are marginal and/or intralesional.

We acknowledge limitations commonly attributed to the retrospective study design, mainly information bias. The analysis was constrained by the records of deceased patients belonging to other counties being unavailable and excluded from analysis, resulting in a modest study cohort for our single institution. The number of recurrences as well as the time‐to‐event may therefore have been other than presented. Regardless, these findings affirm negative margins as the determining factor for local disease recurrence. No reduction in LR was attributed to RT, but this may have been inhibited by the sizing of this cohort and the findings should be interpreted with caution. However, this study represents a diverse population with low‐ and high‐grade lesions at various anatomical sites and both planned and inadvertent surgery. There were no alterations to the management practices during the inclusion period that could have impacted on the oncological outcomes seen in the present study.

## 5 Conclusion

Negative surgical margins continue to be the cornerstone of curative management for MFS and proactive surgical management may be required to lessen morbidity rates. RT did not demonstrate any suppressive effect on LR.

## Synopsis

Optimal resection margins for myxofibrosarcoma remain uncertain, and the role of radiotherapy is debated. This study explores the relationship between surgical margins, radiotherapy, and local recurrence, showing that wide margins are most important for local control, while radiotherapy does not significantly reduce recurrence risk.

## Data Availability

The data that support the findings from this study are available from the corresponding author upon reasonable request. The data are not publicly available due to privacy or ethical restrictions.
